# Unique and late onset infections after Dynamic Dynesys Stabilization in the lumbosacral spine: A retrospective cohort study

**DOI:** 10.1097/MD.0000000000050352

**Published:** 2026-08-21

**Authors:** Po-Hsin Lin, Ting-Wei Chang, Zhuo-Hao Liu, Nan-Yu Chen, Jyi-Feng Chen

**Affiliations:** a Department of Neurosurgery, Chang Gung Memorial Hospital, Chang Gung University, Linkou, Taiwan; b Division of Infectious Diseases, Department of Internal Medicine, Chang Gung Memorial Hospital, Chang Gung University, Linkou, Taiwan.

**Keywords:** dynamic stabilization, infection

## Abstract

This study investigates a unique pattern of late-onset infections occurring exclusively in patients treated with the Dynamic Dynesys Stabilization (DDS) system for degenerative lumbosacral disease. Between 2013 and 2019, 571 patients underwent lumbosacral surgery using the DDS system. A total of 431 patients completed a minimum 2-year follow-up (mean: 5.3 years; range: 2.3–8.9 years). Data on late-onset infections (occurring > 6 months postoperatively) were analyzed, focusing on clinical presentations, comorbidities, radiological findings, and microbiological results. Nine patients (2.1%) developed late-onset infections at a mean interval of 32.5 months after the index surgery. Most affected patients (77.8%) had chronic comorbidities such as diabetes or chronic kidney disease. Common symptoms included low back pain (7/9, 77.8%), fever (3/9, 33.3%), and radiculopathy (2/9, 22.2%). Diagnosis was often delayed (mean: 7.3 weeks) due to indolent symptoms. Notably, screw loosening was observed in 100% of infected cases (9/9). Cultures frequently showed no growth (7/9), with *Propionibacterium* and *Staphylococcus epidermidis* identified in 2 cases. All patients recovered following implant removal and antibiotic therapy. Late-onset infection is an underrecognized complication of the DDS system, primarily affecting patients with chronic comorbidities. While the incidence was low, screw loosening was consistently observed in all infected cases. Although this finding requires validation in comparative studies, it may represent a potential clinical warning sign warranting further evaluation for deep peri-prosthetic infection. Prolonged clinical surveillance is recommended for this high-risk population.

## 1. Introduction

Lumbar fusion surgery remains the standard treatment for degenerative lumbosacral diseases refractory to conservative management.^[1-5]^ While rigid instrumentation provides stability, it is often associated with adjacent-segment degeneration (ASD) and persistent back pain, particularly in long-segment fixations and elderly patients.^[6]^ To address these limitations, dynamic stabilization systems, such as the Dynesys Dynamic Stabilization (DDS) system, were introduced to preserve segmental motion and optimize load sharing across the facet joints.^[7]^

The DDS system utilizes titanium alloy cannulated screws connected by an elastic synthetic compound, offering a non-fusion alternative that potentially reduces the risk of ASD.^[8,9]^ Although mid-term results have demonstrated favorable clinical outcomes in functional scores and pain reduction, some studies remain inconclusive regarding the system’s protective effect on adjacent segments.^[10-12]^ Therefore, the long-term safety profile regarding late-onset complications remains a subject of ongoing clinical surveillance.^[13]^

While acute surgical site infections (SSI) following adult spinal fusion are well-documented, occurring in 0.7% to 12.0% of cases,^[14-16]^ the economic burden of such complications warrants robust evidence-based care.**^[17]^** However, late-onset infections associated with dynamic implants present a unique diagnostic challenge.^[18,19]^ These infections often manifest years after the index surgery with indolent symptoms, potentially linked to the specific design of the implant system.

In this study, we present a single-surgeon series of 571 lumbosacral stabilization procedures. We specifically focus on a distinct pattern of late-onset peri-prosthetic infections observed during a long-term follow-up of up to 10 years. By analyzing the clinical course and radiological markers of these cases, we aim to provide insights into the management and early detection of this late-stage complication.

## 2. Materials and methods

### 2.1. Study design and patient selection

This retrospective, single-center, observational study evaluated the long-term safety of the Dynesys Dynamic Stabilization (DDS) system (Zimmer Spine, Minneapolis). The study protocol was approved by the Institutional Review Board of Chang Gung Memorial Hospital, Linkou, Taiwan (IRB No. 202601342B0). Between 2013 and 2019, 571 consecutive patients underwent lumbosacral stabilization using the DDS system.^[7]^ However, 140 patients were excluded from long-term analysis because they did not complete the mandatory follow-up protocol. Reasons for loss to follow-up included complete symptom relief with refusal of routine return visits, patient relocation, or non-spine-related mortality. Consequently, a total of 431 patients were included in the final analysis. All surgeries were performed by a single senior surgeon and his clinical team to ensure procedural consistency.

The inclusion criteria were: symptomatic degenerative lumbosacral disease (including spinal stenosis, degenerative discopathy, or spondylolisthesis) refractory to conservative treatment and treatment with DDS or Dynesys–Transition–Optima (DTO) hybrid stabilization.^[8,9]^ The exclusion criteria were: preoperative spinal infection; follow-up duration of <24 months; and surgery indicated for acute spinal trauma or primary spinal malignancy.

### 2.2. Clinical and radiological follow-up protocol

All 431 patients maintained regular follow-up at 3 to 6-month intervals for at least 2 years. After the initial 2-year period, patients were followed annually or upon the emergence of new symptoms. The mean follow-up duration for this cohort was 5.3 years (range: 2.3–8.9 years).

### 2.3. Definition and diagnosis of late-onset infection

Late-onset infection was defined as a deep peri-prosthetic infection occurring more than 6 months postoperatively, which was considered clinically distinct from the index surgical procedure. Patients presenting to the emergency room (ER) or outpatient department (OPD) with symptoms such as recalcitrant back pain, fever, or localized wound swelling were screened.

The diagnostic workup included inflammatory markers (white blood cell count, C-reactive protein [CRP], and erythrocyte sedimentation rate [ESR]). Radiological evaluation consisted of plain radiographs to assess for screw loosening or fracture,^[11,20]^ and gadolinium-enhanced magnetic resonance imaging (MRI) to confirm the presence of peri-prosthetic abscess, spondylitis, or psoas muscle abscess.

### 2.4. Data collection and statistical analysis

For patients with confirmed late-onset infection, comprehensive data were collected, including: patient demographics and chronic comorbidities (e.g., diabetes mellitus, chronic renal disease); primary surgical details (operative time, blood loss, and levels of instrumentation); clinical presentation and duration of symptoms; and microbiological results from blood or wound cultures.

## 3. Results

Among the 571 patients who underwent DDS stabilization, no deep wound infections were observed within the initial 6-month postoperative period. However, minor superficial wound infections occurred in several patients, which were successfully managed with local wound care and oral antibiotics. Of the total cohort, 431 patients completed a long-term follow-up of at least 2 years (mean: 5.3 years; range: 2.3–8.9 years). Late-onset infections (occurring > 6 months post-surgery) were identified in 9 patients, resulting in an incidence rate of 2.1% in the long-term follow-up group.

The infected cohort consisted of 8 males and 1 female, with a mean age of 62.3 years (range: 49–76 years). Seven of the 9 patients (77.8%) presented with at least 1 chronic comorbidity, including diabetes mellitus (4/9, 44.4%), hypertension (5/9, 55.6%), and chronic kidney disease (1/9, 11.1%). Detailed clinical data are summarized in Tables 1 and 2.

**Table 1:** Summary of late-onset infection cases

Patient data		Mean/percentage
Male/female	8:1	
Age (yr)	49–76	62.3
Chronic disease (diabetes, chronic kidney disease, rheumatology disease, cancer history, repeat deep wound infections)	7	77.8%
OP time (min)	240–376	270
Blood loss (mL)	50–750	277.8
Levels of implants	3–6	4.3
Symptoms		
Back pain	7	77.8%
Radiculopathy	2	22.2%
Fever	3	33.3%
Wound bulging mass	2	22.2%
Wound disruption	0	0%
Elevation of serum WBC/CRP/ESR	3/7/8	33.3%/77.8%/88.9%
Time length of removal (mo)	10.07–66.96	32.54
Psoas muscle abscess	3	34%
Peri implant abscess	8	89%
Total screw	78	
Screw loosening	22	28.2%
Screw fracture	5	6.4%
Pathogen: no growth	7	77.8%
*Staphylococcus epidermidis*	1	11.1%
*Propionibacterium*	1	11.1%

CRP = C-reactive protein, ESR = erythrocyte sedimentation rate, OP = operation, WBC = white blood cell.

**Table 2:** Individual patient clinical data

Patient number	1	2	3	4	5	6	7	8	9
Gender	F	M	M	M	M	M	M	M	M
Age	64	69	49	54	53	76	56	76	64
BMI	25.6	26.8	28.5	19.6	28.1	22	24.2	24.8	31.4
Comorbidity	HTN, DM, psoriasis	HTN, DM, Rectal villus adenoma	HTN, HBV, hepatic hemangioma	DM		HTN, Gastric cancer	DM	HTN DM CKD COPD	
Skin lesion	Rt ankle ulcer				Bilateral palm and sole hyperpigmentation				Leg wound with repeat Infection
Primary OP detail									
Surgical level	6	3	4	4	5	4	4	3	6
Blood loss (c.c)	50	150	450	100	750	100	250	300	350
OP time (min)	339	312	366	281	246	240	249	260	256
Infection sign									
Back pain	+	+	+	−	+	+	+	+	−
Radiculopathy	−	−	−	−	+	−	−	+	−
Fever	-	+	-	+	-	-	-	+	-
Symptoms duration before diagnosis (d)	7	24	3	4	44	76	15	196	220
Superficial wound infection	−	−	−	−	−	−	−	−	−
Abscess location									
Deep, peri-prothesis	+	+	+	−	+	+	+	+	+
Psoas muscle	−	+	−	+	−	−	−	−	+
Screw loosen	+	+	+	+	+	+	+	+	+
Screw fracture	−	−	+	+	−	−	+	+	+
Pathogen	No growth	*Propioni-bacterium*	No growth	No growth	No growth	No growth	*Staphylococcus epidermidis*	No growth	No growth
Interval following primary surgery (mo)									
Diagnosis	38.54	34.61	24.43	42.61	8.82	13.82	66.83	11.79	47.21
Reoperation	38.75	34.75	24.64	42.75	10.07	14.86	66.97	–	47.25
Lab data									
CRP (mg/L) (elevation)	28.5 (+)	238 (+)	153.9 (+)	92.6 (+)	11.3 (+)	3.1 (−)	2.03 (−)	25.2 (+)	76.5 (+)
ESR (mm/h) (elevation)	64 (+)	104 (+)	98 (+)	101 (+)	54 (+)	82 (+)	10 (−)	121 (+)	92 (+)
Leukocyte count/L (elevation)	8.7k (−)	9.7k (−)	9.7k (−)	11.4k (+)	4.9k (−)	7.1k (−)	11k (+)	9.2k (−)	13.2k (+)

BMI = body mass index, CKD = chronic kidney disease, COPD = chronic obstructive pulmonary disease, CRP = C-reactive protein, DM = diabetes mellitus, ESR = erythrocyte sedimentation rate, HBV = hepatitis B virus, HTN = hypertension, OP = operation.

The predominant clinical symptom was low back pain (7/9, 77.8%), followed by fever (3/9, 33.3%) and radiculopathy (2/9, 22.2%). The interval from symptom onset to confirmed diagnosis averaged 55.7 days overall. Patients presenting to the Emergency Department were diagnosed significantly earlier (mean: 23.3 days) compared to those with indolent symptoms seen in the outpatient clinic (mean: 78.6 days).

**Figure 1.**
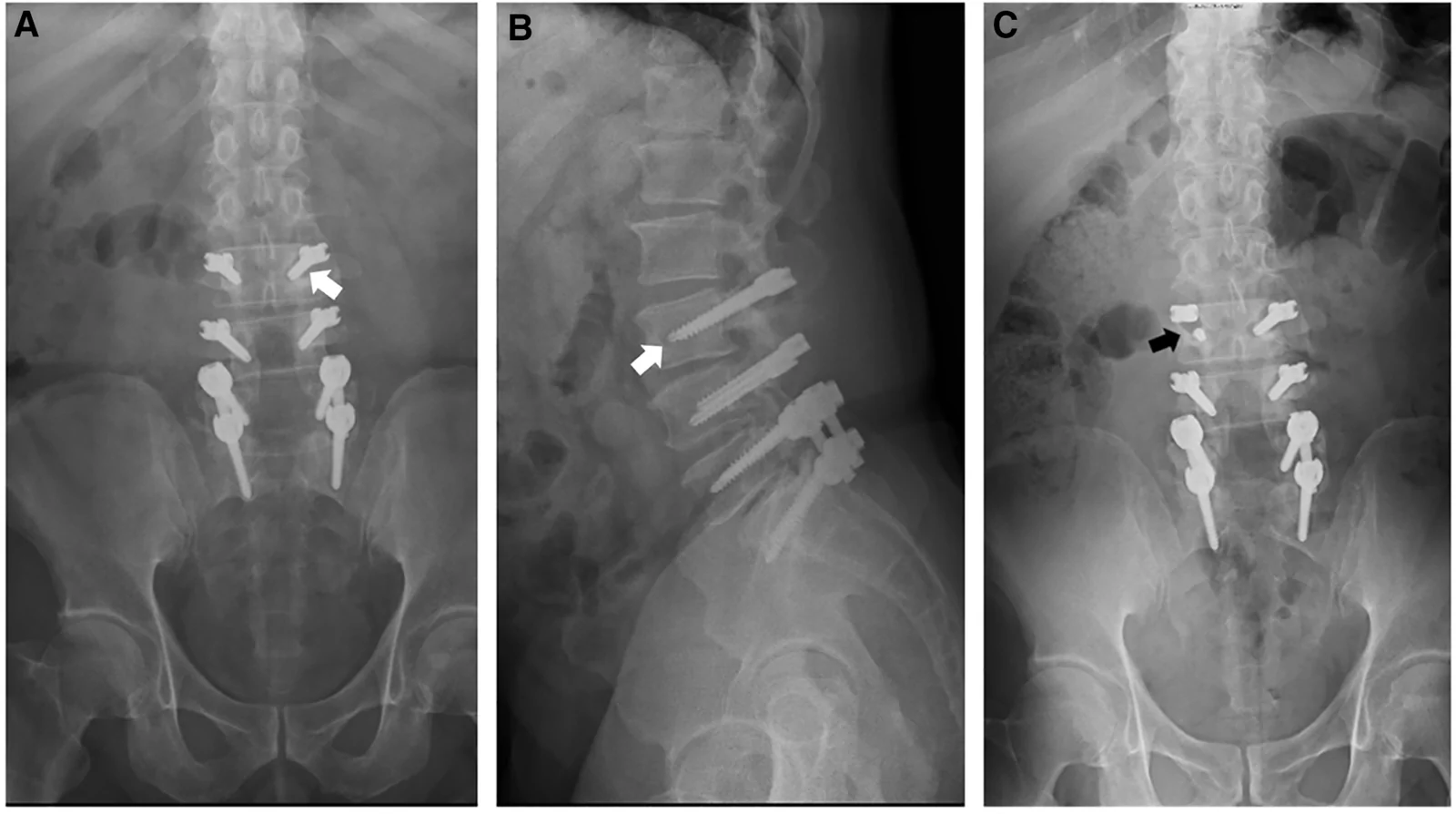
Radiographic findings in patients with late-onset infection after Dynesys Dynamic Stabilization. (A) Anteroposterior and (B) lateral plain radiographs showing pedicle screw loosening (white arrow) and mechanical instability of the dynamic stabilization construct. (C) Follow-up anteroposterior radiograph demonstrating a progressive screw fracture (black arrow), raising suspicion for deep peri-prosthetic infection.

**Figure 2.**
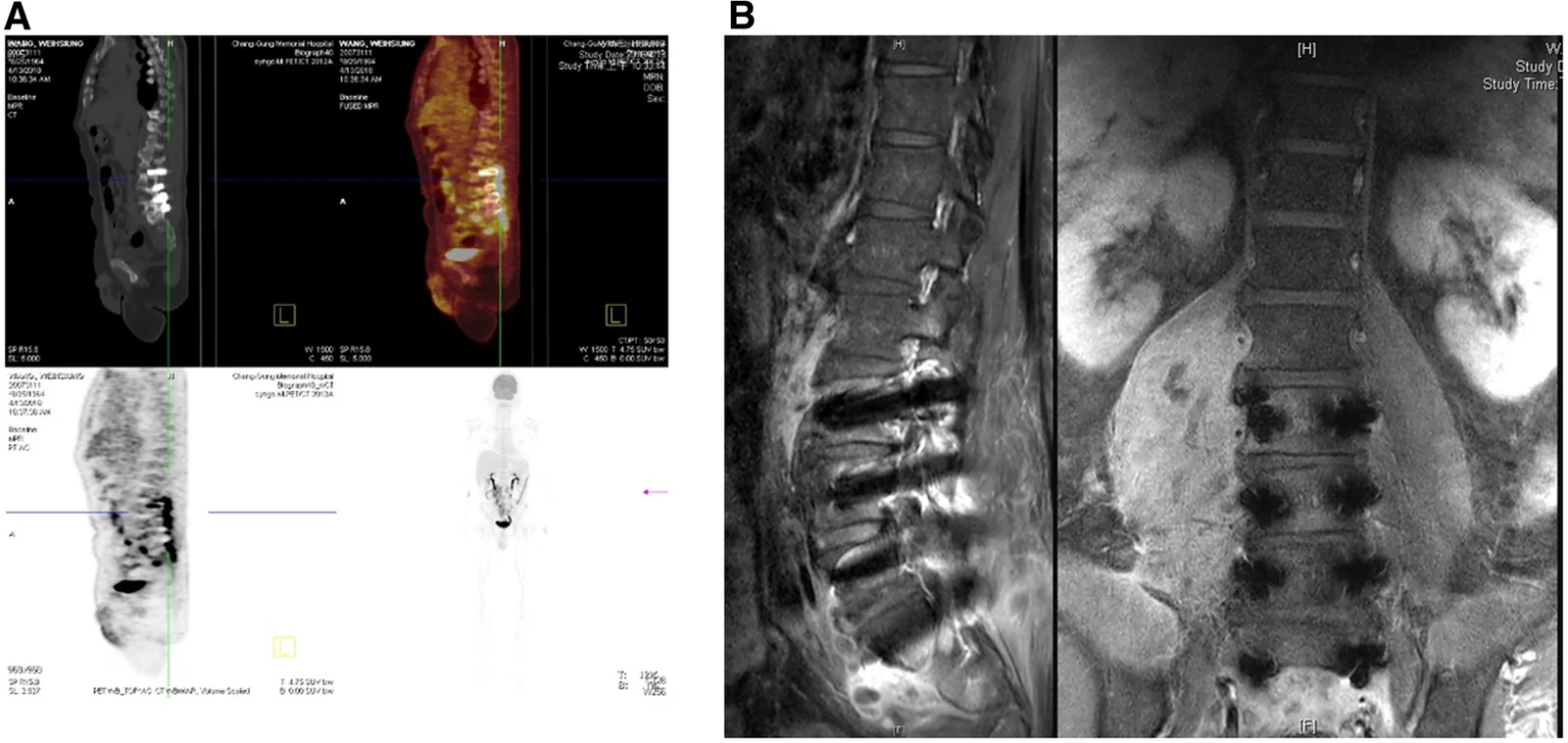
Advanced imaging findings of late peri-prosthetic infection associated with the DDS system. (A) PET/CT longitudinal and coronal reconstructions showing intense, localized radiotracer hypermetabolism surrounding the lumbosacral instrumentation, indicative of active peri-prosthetic infection. (B) Gadolinium-enhanced magnetic resonance imaging (MRI) showing extensive peri-prosthetic inflammatory change with associated paraspinal and deep soft tissue abscess formation around the stabilized lumbar segments. CT = computed tomography, DDS = Dynesys Dynamic Stabilization, MRI = magnetic resonance imaging, PET = positron emission tomography.

Laboratory evaluation upon diagnosis revealed elevated C-reactive protein (CRP) in 7/9 patients (77.8%) and elevated erythrocyte sedimentation rate (ESR) in 8/9 patients (88.9%). Radiographic assessment demonstrated pedicle screw loosening in all 9 patients (100%), and screw fractures were observed in 5 patients (55.6%). Such radiographic signs of loosening and fracture are documented indicators of mechanical failure or potential low-grade infection in dynamic systems.^[20,21]^ Typical case examples are shown in Figures 1 and 2.

MRI findings confirmed deep peri-prosthetic abscesses in 8 patients (88.9%), with 3 patients (33.3%) also exhibiting iliopsoas muscle abscesses. Microbiological analysis of blood and wound cultures showed no growth in 7 patients (77.8%). Identified pathogens included *Propionibacterium* (n = 1) and *Staphylococcus epidermidis* (n = 1).

Instrumentation removal and prolonged antibiotic therapy were performed in 8 patients, all of whom achieved favorable clinical outcomes. One patient (case 8) was managed solely with antibiotics due to poor general condition and showed symptomatic improvement.

## 4. Discussion

The DDS system represents a significant shift from rigid fixation toward motion-preserving philosophies. As a non-fusion, transpedicular screw-based construct, DDS utilizes flexible interconnecting components to allow for constrained yet functional motion within the functional spinal unit (FSU). The primary biomechanical objective of this system is to maintain segmental kinematics while effectively unloading the facet joints – a critical factor in preventing degenerate changes.^[8]^ Many short-term and mid-term follow-up studies have shown good results for the use of Dynesys and/or the DTO system^[7,9]^ although some studies have inconclusive results in proving restoration of physiologic weight balance and protective effect on ASD.^[10-12]^

Surgical site infection (SSI) following spinal fusion remains a major clinical concern, with reported rates ranging from 0.7% to 12.0% depending on surgical complexity and patient comorbidities.^[14-16]^ Unlike acute surgical site infections that occur within 30 days and are typically attributed to intraoperative contamination, late peri-prosthetic infections require heightened long-term surveillance and a different diagnostic framework. Our 2.1% late infection rate falls within the lower range of published SSI rates for adult spinal fusion (0.7–12.0%),^[14-16]^ suggesting that the DDS system itself does not confer an unusually elevated infection risk, but that infections which do occur may manifest unusually late.^[17]^ In our cohort, we observed similar late infectious complications associated with the DDS and/or DTO system, prompting this detailed clinical analysis and literature review.

In our series, no acute postoperative SSIs occurred within the initial 6 months, which may be attributed to standardized sterile techniques and meticulous intraoperative wound irrigation. However, 9 patients developed late-onset infections (>6 months) at a mean interval of 32.5 months, yielding an incidence of 2.1% in our long-term cohort. This delayed manifestation aligns with findings by Lutz et al, who documented late Dynesys infections at a median of 52 months. In contrast to Lutz et al^[19]^, however, our overall infection rate was considerably lower (2.1% vs 22%), likely reflecting differences in patient comorbidities, case volume, and perioperative management. In our series, low back pain was the predominant clinical symptom (77.8%), followed by fever (33.3%), radiculopathy (22.2%), and a palpable wound mass (22.2%). Four patients (patients No. 2, 3, 4, and 7) presented acutely to the emergency department, whereas the remaining 5 were evaluated in the outpatient clinic. Notably, none of the patients exhibited localized erythema or wound dehiscence upon initial presentation. Reviewing the literature, the reported risk factors for SSI after adult spinal fusion surgery include blood loss over 1 liter, longer surgeries (>5 hours), previous spine surgeries and/or surgical site infections, obesity, and diabetes.^[14-16]^ In our patients, not only diabetes was noted. There were also patients with a history of cancer, chronic kidney disease, rheumatologic disease, steroid use, and a history of deep wound infections in the limbs.

In 1 comprehensive review, the overall reported infection rate among patients who used the DSS was 4.3%.^[18]^ According to another study, the infection rate in long levels (>5) was up to 62.5 percent higher than in short segments.^[22]^ Fixation extent (ranging from 3 to 6 levels) showed no apparent impact on late infection rates in our study.

All of our patients were followed with imaging when they arrived at the ER or OPD due to late infection symptoms. Notably, all of the patients in our studies were diagnosed with screw loosening following the imaging study, which is more common than in the prior review. Previous studies demonstrate that the incidence of screw loosening in DSS is 3% to 4%.^[7,21]^ Although the radiographic finding of screw loosening can be problematic, some studies have shown that overall outcomes are similar regardless of screw issues. They found similar results on the visual analog scale for low-back pain and the Oswestry Disability Index for functional disability, regardless of screw loosening.^[11,20]^ However, Bothmann and colleagues suggested that the ongoing back and leg pain experienced by their seven patients with screw loosening was directly related to the surgical hardware.^[23]^ There are additional causes of implant/screw loosening besides infection (i.e., biomechanical as well as chemical).^[24]^ Apart from spinal fusion implants, septic loosening is a serious side effect that occurs after knee and hip replacements.^[25]^ The size of the implant, in particular, and the surface area available for biofilm formation are widely recognized risk factors for implant infection. The Dynesys system has higher long-term repetitive demands on mechanical strength and construct durability because there is no rigid fusion. Pedicle screw loosening and fracture are symptoms of this, indicating an abnormally high range of motion in the stabilized lumbar spine segments. Furthermore, 1 study further hypothesized that symptomatic screw loosening might be linked to a low-grade infection, drawing this conclusion based on the findings from microbiological culture analyses.^[26]^ Consequently, the biomechanical characteristics of the DDS system may contribute to an increased risk of screw loosening when deep peri-prosthetic infection occurs. Based on this observation, we hypothesize that radiographic screw loosening may serve as a potential clinical warning sign prompting further diagnostic evaluation for deep infection. However, this hypothesis requires prospective validation with adequate comparator groups, including noninfected DDS patients with screw loosening, before it can be considered a reliable diagnostic criterion.

This research acknowledges several limitations. First, the absence of a comparator group of noninfected DDS patients with screw loosening is the most critical constraint: without this comparator, it is not possible to determine the specificity of screw loosening as an indicator of infection, nor to calculate its diagnostic performance (sensitivity, specificity, or positive predictive value). Second, microbiological confirmation was achieved in only 2 of 9 cases (22.2%), with 7 cultures showing no growth. This high culture-negative rate represents a well-recognized diagnostic challenge in late-onset spinal instrumented infections.^[27]^ The extensive surface area of nonmetallic synthetic components in the DDS system facilitates bacterial biofilm formation. Additionally, indolent, low-virulence organisms such as *Propionibacterium* and prior antibiotic administration before presentation likely contributed to negative routine cultures. Future studies should employ sonication of explanted hardware and prolonged culture protocols to improve microbiological yield. Third, the 24.5% attrition rate (140/571 patients lost to long-term follow-up) introduces potential selection bias. If asymptomatic patients were more likely to discontinue follow-up, our reported 2.1% late infection rate might slightly overestimate the true incidence. To validate these findings, a comprehensive prospective multicenter investigation with standardized diagnostic criteria, systematic microbiological protocols, and an adequately powered comparator cohort would be necessary.

## Author contributions

**Conceptualization:** Jyi-Feng Chen.

**Data curation:** Po-Hsin Lin, Ting-Wei Chang, Zhuo-Hao Liu, Nan-Yu Chen, Jyi-Feng Chen.

**Formal analysis:** Ting-Wei Chang, Nan-Yu Chen.

**Investigation:** Nan-Yu Chen.

**Methodology:** Ting-Wei Chang, Zhuo-Hao Liu.

**Project administration:** Po-Hsin Lin, Ting-Wei Chang.

**Supervision:** Zhuo-Hao Liu, Nan-Yu Chen.

**Validation:** Zhuo-Hao Liu, Jyi-Feng Chen.

**Writing** – **original draft:** Po-Hsin Lin, Ting-Wei Chang, Zhuo-Hao Liu.

**Writing** – **review & editing:** Jyi-Feng Chen.
